# Galectin-9 as a new biomarker of acute-on-chronic liver failure

**DOI:** 10.1038/s41598-024-73397-6

**Published:** 2024-09-27

**Authors:** Jun Ling, Shaoli You, Weiwei Chen, Xinxin Yang, Yiwen Xv, Bing Zhu

**Affiliations:** 1 Hepatology Department, The Fifth Medical Center of Chinese PLA General Hospital, No. 100, Xisi Huanzhong Road, Beijing, 10039 China; 2 Infectious Disease Department, The Fifth Medical Center of Chinese PLA General Hospital, Beijing, 10039 China

**Keywords:** Gal-9, ACLF, Prognosis, Clinical diagnosis, Hepatology, Hepatitis, Liver diseases

## Abstract

Galectin-9 (Gal-9) expression in patients with acute-on-chronic liver failure and its correlation with prognosis remain unclear. This study investigated the relationship between liver failure prognosis and Gal-9 expression analysis in patients with acute-on-chronic liver failure. Patients with acute-on-chronic liver failure attributable to hepatitis B and those with chronic hepatitis B were included in this single-center prospective cohort study. The Gal-9 levels in the acute-on-chronic liver failure group were significantly higher than those in the chronic hepatitis B group, and there was an upregulation of Gal-9 and T-cell immunoglobulin domain and mucin domain-3 expressions in peripheral blood T cells. Gal-9 was localized in the regenerative areas of liver tissues in patients with acute-on-chronic liver failure, co-localizing with Kupffer cells. Kaplan–Meier survival curves showed that patients with Gal-9 levels < 9.6 ng/ml had a worse prognosis, with the area under the receiver operating characteristic curve (AUC-ROC) being similar to that of the Model for End-Stage Liver Disease score. The combined ROC curve of the two had better predictive performance, with an AUC of 0.945. High Gal-9 levels in liver regenerative areas can serve as a prognostic marker, indicating a better prognosis for patients with hepatitis B virus-acute-on-chronic liver failure.

**Supplementary Information:**

The online version contains supplementary material available at 10.1038/s41598-024-73397-6.

## Introduction

Hepatitis B virus (HBV)-associated acute-on-chronic liver failure (ACLF) is characterized by the acute deterioration of liver function in patients with chronic liver disease. It is the most prevalent type of liver failure in China and manifests as a syndrome of multiorgan dysfunction involving intrahepatic and extrahepatic functions. A comprehensive internal medicine treatment plan frequently results in a poor prognosis and a high likelihood of short-term mortality. In China and other Asian nations, the main cause of ACLF is chronic hepatitis B (CHB). Currently, there is no proven cure for ACLF. Therefore, enhancing clinical outcomes for individuals with ACLF requires early identification and a correct prognosis^1^.

Galectin-9 (Gal-9) is a member of the galectin family that specifically recognizes and binds to lactoside. It is primarily involved in cell proliferation, differentiation, adhesion, aggregation, and apoptosis and is widely expressed in bodily tissues^2,3^. Gal-9 is involved in many diseases and functions in innate and acquired immunity, contributing to immune homeostasis maintenance in the liver. The Gal-9-induced physiological changes are associated with its intracellular and extracellular concentrations, its binding to neighboring cells, and the signals emitted after binding to cell surface receptors^4,5^. Gal-9’s ligand, Tim-3 (T-cell immunoglobulin domain and mucin domain-3), is thought to be an immune checkpoint receptor that controls innate and adaptive immunities to maintain homeostasis^6^. Gal-9-induced Tim-3 activation can suppress effector T-cell activity and adversely affect the T helper 1 cell immunological response^7^.

According to a recent study, Gal-9 is a noninvasive biomarker that is easily quantifiable, dependable, and sensitive. Gal-9 might have conflicting biological effects, acting as a “double-edged sword,” with opposing biological outcomes while maintaining liver immune homeostasis, depending on the source, target cells, and disease progression. The role of Gal-9 in chronic liver disease is unclear, and studies on its potential prognostic significance are scarce. While Gal-9 was mostly expressed in tumor cells in two investigations, it was also significantly expressed in Kupffer cells (KCs) in Li et al.’s study. Furthermore, conflicting results have been reported about Gal-9’s prognostic significance in patients with hepatocellular cancer. Gal-9 expression levels have been linked to a poor prognosis in patients with acute liver failure caused by drug-induced liver injury, according to Rosen et al.^8^ However, there are no studies on Gal-9 expression in patients with ACLF or its correlation with prognosis; thus, further research is warranted.

Therefore, this study aimed to detect Gal-9 expression in the cells and plasma of patients with HBV-ACLF using flow cytometry and enzyme-linked immunosorbent assay (ELISA), as well as the levels of plasma inflammatory factors, to ascertain the relationship between Gal-9 expression and the prognosis and outcome of patients with HBV-ACLF. The clinical importance of Gal-9 as a biomarker for HBV-ACLF diagnosis and its potential as a prognostic predictor of HBV-ACLF were also investigated in this study.

## Methods

### Ethics statements

The local ethics committee approved the study protocol (approval number: KY-2022-6-44-2), and the research was performed in compliance with the ethical principles of the 1975 Declaration of Helsinki (6th revision, 2008). Informed consent was obtained from the patients or their first relatives if they could not provide informed consent themselves.

### Patient recruitment

Thirty-three patients diagnosed with ACLF and 33 diagnosed with CHB were hospitalized at a local medical center between March 2023 and October 2023 and were prospectively selected. Inclusion criteria were age 16–70 years, any sex, and diagnosis of ACLF or CHB according to the criteria defined by the Asian Pacific Association for the Study of the Liver (APASL)^9^. Additional liver tissue samples, obtained from 15 patients with previous liver failure undergoing liver transplantation, were selected for tissue analysis.Exclusion criteria for patients with ACLF were concomitant diabetes; hyperthyroidism; severe heart, brain, kidney diseases, or other systemic diseases; malignant tumors or active gastrointestinal bleeding; chronic liver failure; and poor compliance. Exclusion criteria for patients with CHB were chronic hepatitis of other causes; liver damage caused by drugs, toxins, or metabolic factors; or continuous hepatitis B surface antigen positivity due to causes other than viral hepatitis. After confirmation of the diagnosis, blood samples were collected from all patients early in the morning on the first day of admission for laboratory and assay testing, followed by a collection of the patients’ first blood test results for analysis. Prospective cases were followed for 90 days.

### Blood sampling

For the trials, a total of 10–12 mL of blood was collected in tubes containing ethylene diamine tetraacetic acid. For biochemical, serological, and laboratory testing, plasma was extracted from a portion of the blood sample, and aliquots were kept at − 80 °C. From the remaining whole blood samples, peripheral blood mononuclear cells (PBMCs) were separated.

### PBMC isolation

PBMCs were extracted from patient and control blood samples using the Ficoll-Hypaque density gradient centrifugation technique. The separated cells were kept in liquid nitrogen after being frozen in a 90% fetal bovine serum and 10% dimethyl sulfoxide solution overnight at − 80 °C (Supplementary Methods S1).

### Flow cytometry analysis

PBMCs were obtained from fresh peripheral blood using Ficoll-Isopaque gradient centrifugation. Fluorescently conjugated antibodies were used to stain the isolated PBMCs after they had been grown in vitro. Flow cytometry analysis was conducted using the FACSCanto Plus flow cytometer (BD Biosciences, Franklin Lakes, NJ) and the FlowJo VX software (Tree Star, Ashland, OR). The anti-CD45 APC/CY7, anti-7AAD PerCP, anti-CD8 BV421, anti-CD4 PECY7, anti-CD3 BV510, anti-Tim-3 PE, and anti-Galectin-9 APC mice anti-human monoclonal antibodies were employed. The cells were analyzed, fixed, and washed.

### Enzyme-linked immunosorbent assay

Human interferon-gamma (IFN-γ) and human Gal-9 ELISA kits (R&D Systems, Minneapolis, MN) were used. The manufacturer’s instructions were followed in the preparation of the reagents, standard dilutions, and samples. In a microplate shaker, each well received 100 µL of the prepared standard, control, or diluted sample and was incubated for 2 h. Following washing, each well received 200 µL of the conjugate, which was then incubated for an additional 2 h. Following another wash, 200 µL of the substrate solution was added to each well and incubated for 30 min without light. The absorbance at 450 nanometers was measured 30 min after adding 50 µL of the stop solution. Every step was performed at ambient temperature.

### Immunohistochemistry (IHC) staining

To evaluate Gal-9 expression, representative Gal-9-stained regions were identified under low magnification (4 ×), and Gal-9 expression was observed semi-quantitatively under high magnification (20 ×) (Supplementary Methods S1).

### Immunofluorescence staining

Immunofluorescence staining was performed using Gal-9-specific antibodies (AF2045, R&D Systems; 1:400) and CD68 antibodies (MAB20401, R&D Systems; 1:2000) on slides. The Supplementary Methods S1 provide step-by-step instructions for immunofluorescence staining.

### Statistical analyses

Prism (version 9.01; GraphPad, La Jolla, CA) and SPSS (version 25; IBM Corp., Armonk, NY) were used to perform statistical analyses. Normal data were compared using the t-test, and non-normal data were compared using the Mann–Whitney U-test. Results were presented in quartiles; unpaired t-tests were used to detect differences in levels between groups. Correlation heatmaps, as well as survival analyses, were generated using R software (Statistical Reporting Project, Vienna, Austria). Cutoff values were selected using the surv_cutpoint function in the survminer package in R. Quantitative analysis of immunohistochemistry and immunofluorescence was performed using Image J. Statistical significance was set at a p-value of < 0.05.

## Results

### The demographic profile of patients

After follow-up, 33 patients were included in the CHB group and 33 in the ACLF group. The patients’ baseline clinical characteristics are presented in Table 1. Patients with HBV-ACLF showed significantly higher alanine aminotransferase (ALT), aspartate aminotransferase (AST), total bilirubin (TBIL), direct bilirubin (DBIL), alpha-fetoprotein (AFP), white blood cell (WBC), and serum ferritin levels than patients with CHB. However, patients with HBV-ACLF had considerably lower levels of blood platelets, lactic acid (LA), red blood cell (RBC), and prothrombin activity (PTA) than patients with CHB. Age, sex, or creatinine (Cr) level showed no discernible differences between the patient groups.

**Table 1:** Comparison between patients with HBV-ACLF and those with CHB at baseline.

Variable	CHB	ACLF	*p*-value^†^
	(*n* = 33)	(*n* = 33)	
Sex			
Male	28	29	ns
Female	5	4	ns
ALT (U/L)	23 [17, 45.5]	92 [55.50, 264.50]	0.000
AST (U/L)	22.5 [18.00, 37.5]	139 [71.50, 238.00]	0.000
BA	10 [4, 32]	217 [152.5, 260.5]	0.000
TBil (µmol/L)	15.45 [10.50, 19.40]	306 [240.4, 418.25]	0.000
DBil (µmol/L)	5.15 [3.60, 7.10]	230 [178.45, 316.2]	0.000
AFP (ng/mL)	2.42 [1.44, 5.85]	36.26 [12.51, 105.7]	0.000
WBC (×10^9^/L)	4.98 [4.22, 5.99]	7.97 [5.49, 11.59]	0.000
RBC (×10^12^/L)	4.61 [4.12, 4.98]	3.28 [2.98, 3.79]	0.000
Cr	83 [78, 90]	86 [76, 101]	ns
LA	3 [2.73, 4.55]	1.84 [1.29, 2.9]	0.006
CRP	1.2 [0.5, 1.9]	9.6 [6.45, 19.35]	0.000
PCT	0.033 [0.021, 0.114]	0.66 [0.33, 0.95]	0.000
INR	1.04 [0.99, 1.07]	1.9 [1.72, 2.15]	0.000
SF (ng/mL)	222.3 [89.87, 531.32]	1354 [496.75, 1735]	0.001
PTA	93.80 [83.05, 108.90]	34.70 [30.10, 40.95]	0.000
			t-test
Age	52 [40.75, 58.00]	49 (40.00, 57.0)	ns
MELD	5.57 [3.63, 7.49]	18.22 (15.5, 19.98)	0.000
PLT (×10^9^/L)	157 [101, 179.5]	73 (49.1, 19.5)	0.000

^†^Analysis was performed using the Mann–Whitney U test or t-test, and statistical significance was set at p values < 0.05.

*ALT* alanine aminotransferase,* AST* aspartate aminotransferase,* PTA* prothrombin activity,* BA* bile acids,* TBIL* total bilirubin,* DBil* direct bilirubin,* AFP* alpha-fetoprotein,* WBC* white blood cell,* RBC* red blood cell,* Cr* creatinine,* LA* lactic acid,* PLT* blood platelet,* SF* serum ferritin,* ns* not significant,* CHB* chronic hepatitis B,* ACLF* acute-on-chronic liver failure.

### Plasma Gal-9 expression is increased in patients with HBV-ACLF

**Fig. 1:**
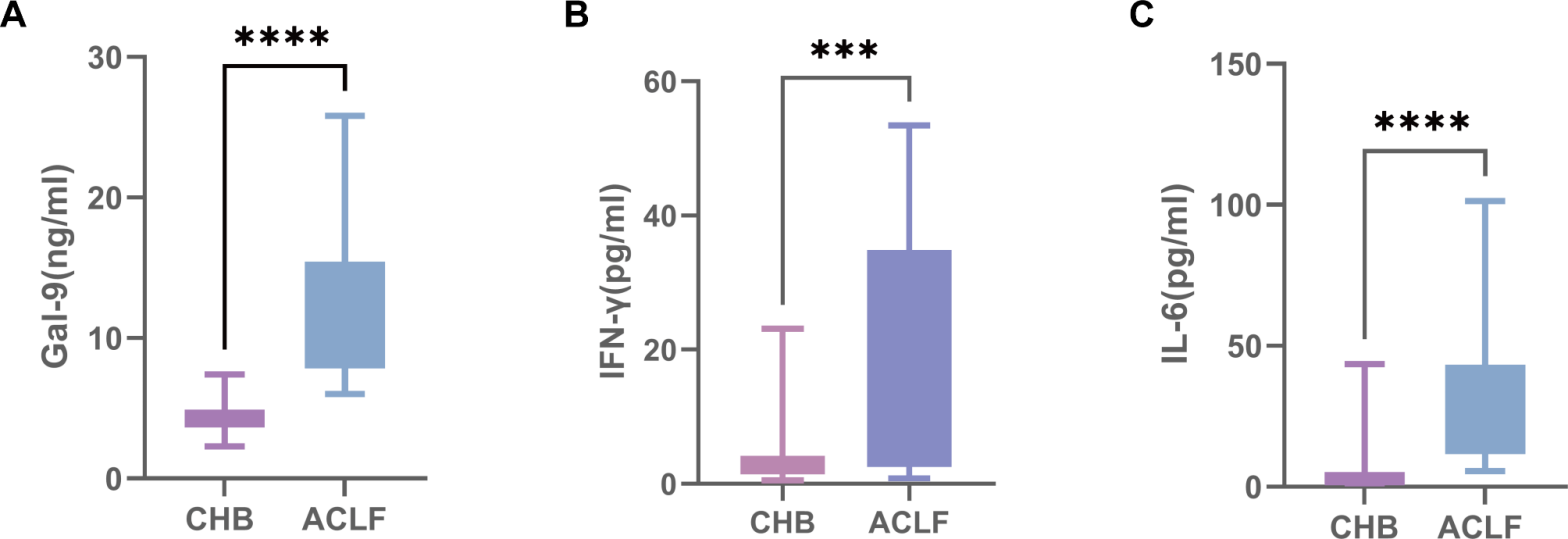
Plasma expression levels of Gal-9, IFN-γ, and IL-6. (**a**) Gal-9 expression is significantly elevated in patients with HBV-ACLF. (**b**) IFN-γ expression is significantly elevated in patients with HBV-ACLF. (c) IL-6 expression is significantly elevated in patients with HBV-ACLF.* Gal-9* galectin-9,* IFN-γ* interferon gamma,* IL-6* interleukin-6,* HBV-ACLF* hepatitis B virus-associated acute-on-chronic liver failure.

Compared with the CHB group’s plasma levels, the HBV-ACLF group’s values of interleukin-6 (IL-6), IFN-γ, and Gal-9 were significantly higher (*p* < 0.0001, *p* = 0.0001, and *p* < 0.00022, respectively) (Fig. 1).

### In patients with HBV-ACLF, CD4 + T/CD8 + T cells express Gal-9 and Tim-3 at higher levels

**Fig. 2:**
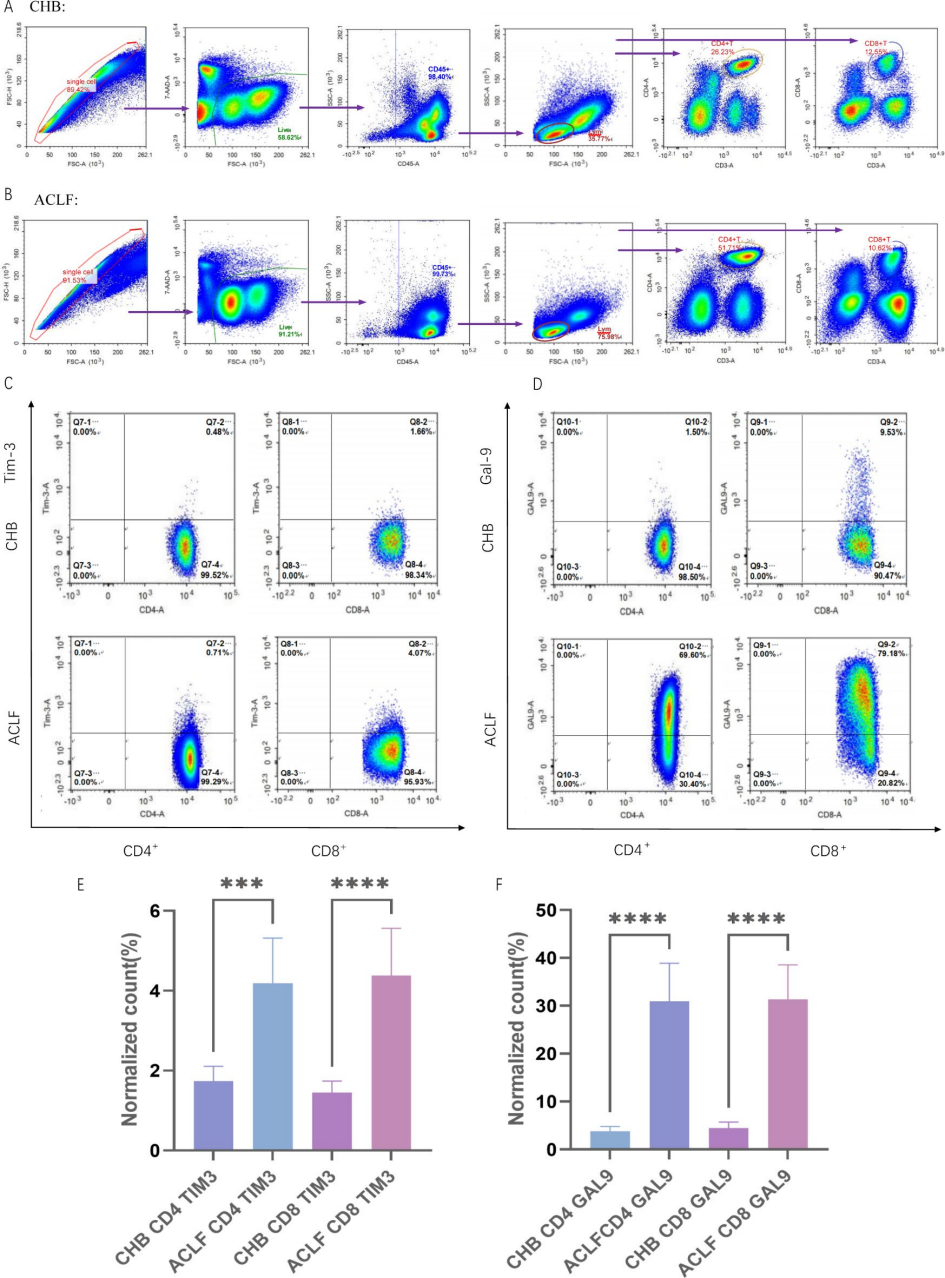
Gal-9 and Tim-3 expressions are upregulated in T cells of patients with HBV-ACLF. (**a**) Flow cytometry gating strategy for patients with CHB. (**b**) Flow cytometry gating strategy for patients with HBV-ACLF. (**c**) Comparison of the number of CD4^+^ T and CD8^+^ T cells expressing Tim-3 in patients with CHB and those with HBV-ACLF. (**d**) Comparison of the number of CD4^+^ T and CD8^+^ T cells expressing Gal-9 in patients with CHB and those with HBV-ACLF. (**e**) Summary statistics of the number of CD4^+^ T and CD8^+^ T cells expressing Gal-9 in patients with CHB and those with HBV-ACLF. (**f**) Summary statistics of the number of CD4^+^ and CD8^+^ T cells expressing Tim-3 in patients with CHB and those with HBV-ACLF. Statistical analysis was performed using the t-test for unpaired data; only significant p-values (*p* < 0.05) are indicated. *Gal-9* galectin-9,* Tim-3* T-cell immunoglobulin domain and mucin domain-3,* HBV-ACLF* hepatitis B virus-associated acute-on-chronic liver failure,* CHB* chronic hepatitis B.

We subjected the PBMCs from both patient groups to flow cytometry to detect Gal-9 and Tim-3 expressions in their CD4^+^ T and CD8^+^ T cells, and the flow cytometry circle-gate paths are shown in Fig. 2a,b. Compared to patients with CHB, individuals with HBV-ACLF had a considerably higher frequency of CD4 + and CD8 + T cells expressing Tim-3 or Gal-9 (Fig. 2e,f). To illustrate particular cell expression, we chose two patients: one with HBV-ACLF and the other with CHB (Fig. 2c,d).

### Expression of Gal-9 is increased in patients with HBV-ACLF

**Fig. 3:**
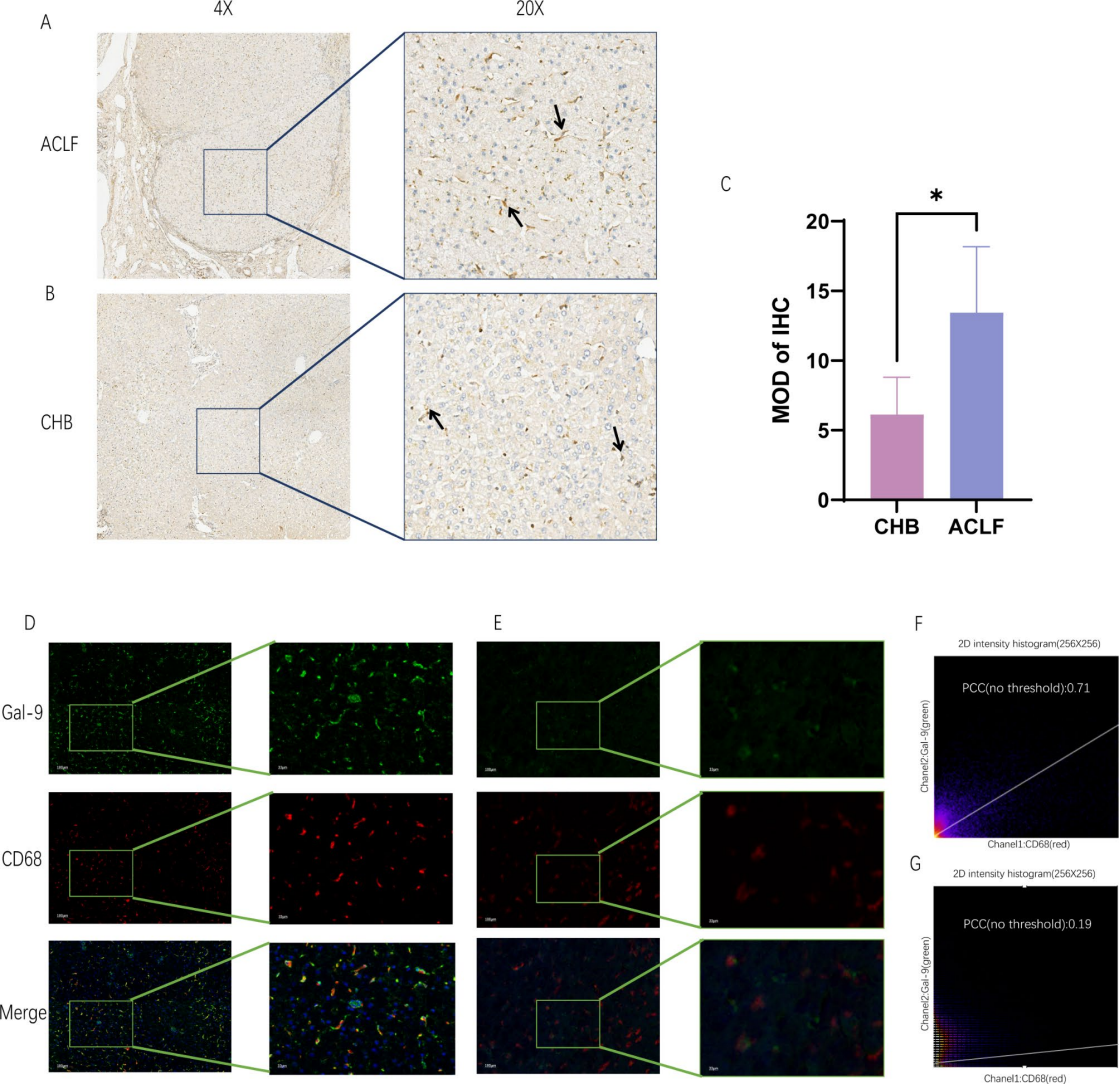
Representative IHC staining results showing the expression of Gal-9 in CHB and ACLF tissues, and dual IF staining results showing the co-localization of Gal-9 with KC cell markers. (**a**) Gal-9 expression in ACLF tissues; (**b**) Gal-9 expression in CHB tissues; (**c**) quantitation of the IHC of Gal-9; (**d**) localization of Gal-9 to CD68 in ACLF green channel: Gal-9; red channel: CD68 and merge; (**e**) localization of Gal-9 to CD68 in CHB (**f**) quantification of the co-localization of Gal-9 and CD68 in ACLF; (**g**) quantification of the co-localization of Gal-9 and CD68 in CHB.* IHC* immunohistochemistry,* Gal-9* galectin-9,* ACLF* acute-on-chronic liver failure,* KC* Kupffer cell,* CHB* chronic hepatitis B.

Samples of liver tissue were obtained from 15 patients with HBV-ACLF and 15 with CHB who underwent liver transplantation and liver puncture, respectively. The immunohistochemical staining results are shown in Fig. 3a,b. Five fields of view were selected from each patient’s specimen using Image pro puls analysis. As seen in Fig. 3c, patients with CHB had a lower Gal-9 level than patients with ACLF, with a p-value of < 0.05, indicating a statistically significant difference. In addition, we found that Gal-9 in patients with ACLF was located in the regenerated cirrhotic nodules.

### Gal-9 is expressed by KCs

To evaluate Gal-9’s possible role in patients with HBV-ACLF in more detail, we used immunofluorescence staining to specifically localize Gal-9 and analyze the cell types in which it was expressed. In patients with ACLF, immune paralysis is a critical aspect of the pathogenic mechanism, and the primary immune cells in the liver are KCs; therefore, we used the KC cell surface marker CD68 to costain with Gal-9 (Fig. 3d,e). Figure 3d shows patients with HBV-ACLF, and Fig. 3e shows patients with CHB. The results revealed that Gal-9 colocalised with CD68 in the liver tissues of patients with ACLF; the quantitative co-localisation results are shown in Fig. 3f. The co-localisation effect was not significant in the liver tissues of patients with CHB (Fig. 3g).

### LGALS9 expression is associated with ACLF indicators

Spearman correlation analysis showed that plasma Gal-9 was positively correlated with ALT, AST, PTA, TBIL, DBIL, AFP, WBC, Cr, ferritin, IFN-γ, INR, and IL-6 values and negatively correlated with LA and RBC values (*p* < 0.05).

**Fig. 4:**
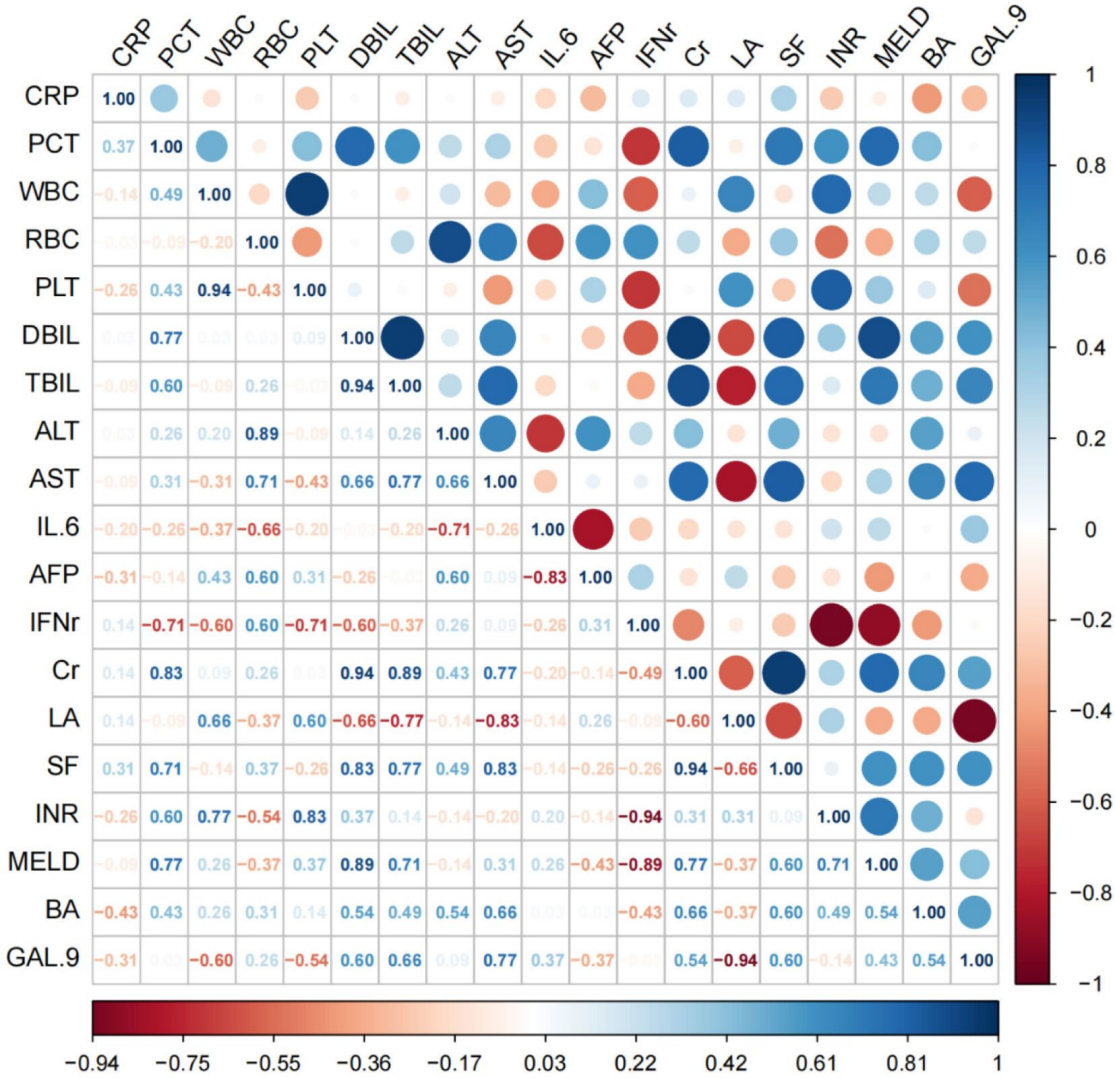
Bubble plot of the correlation between Gal-9 and liver failure indicators: the larger the bubble, the stronger the correlation; blue represents a positive correlation, and red represents a negative correlation.* Gal-9* galectin-9.

Among the commonly analyzed clinical indicators associated with liver failure, we found that Gal-9 had the strongest positive correlation with AST, with a coefficient of 0.77, followed by a positive correlation coefficient of 0.66 with TBIL. The strongest negative correlation was observed with LA, with a coefficient of 0.94. Figure 4 shows a heat map of the correlation matrix.

### Gal-9 levels in patients with HBV-ACLF are correlated with prognosis

**Fig. 5:**
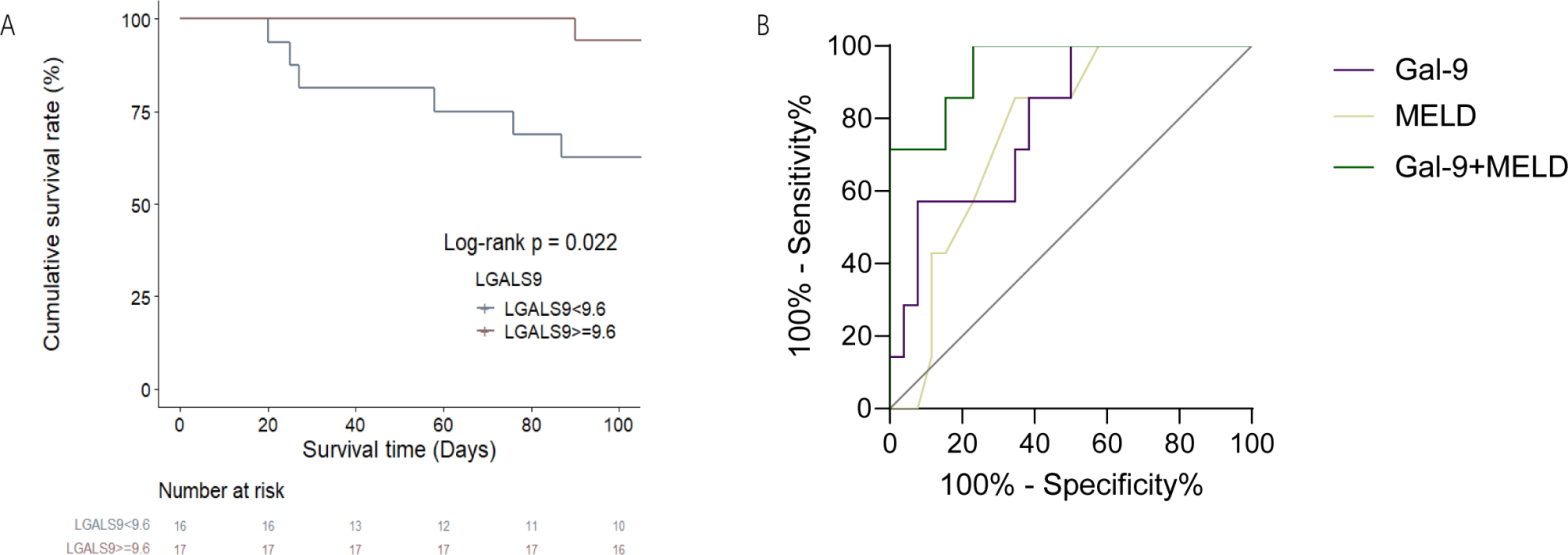
(**a**) Kaplan–Meier survival curve showing the association between Gal-9 expression and overall survival in patients with HBV-ACLF. (**b**) ROC curves for Gal-9 and MELD using values obtained from patients with ACLF. The AUC of Gal-9 is 0.79 (95% CI   0.6251–0.9683; *p* = 0.0174), that of MELD is 0.76 (95% CI  0.5992–0.9338; *p* = 0.0327), and that of Gal-9 + MELD is 0.945 (95% CI  0.863–1.000; *p* = 0.000). *Gal-9* galectin-9,* HBV-ACLF* hepatitis B virus-associated acute-on-chronic liver failure,* ROC* receiver operating characteristic,* MELD* model for end-stage liver disease,* CI* confidence interval,* AUC* area under the curve.

We also assessed Gal-9’s prognostic significance in patients with HBV-ACLF and investigated any correlation between Gal-9 expression and overall survival. The receiver operating characteristic (ROC) curve was applied to find the optimal threshold value of 9.6 ng/ml, and patients in the HBV-ACLF group were stratified according to this value. The survival curves of the patients with HBV-ACLF with different levels of Gal-9 expression were investigated using the Kaplan–Meier technique (Fig. 5a), yielding a p-value of 0.022.

### Gal-9 may serve as a prognostic marker for HBV-ACLF

Since the Model for End-Stage Liver Disease (MELD) is indicative of severe liver disease and commonly used for the assessment of patients with liver failure, it was selected as a reference to assess the predictive ability of Gal-9 for the prognosis of patients with ACLF. We plotted the ROC curves (Fig. 5b) for Gal-9 and MELD based on 90-day survival (death or survival), resulting in an area under the curve (AUC) of 0.76 for MELD and 0.79 for Gal-9. Owing to their similar predictive abilities, we further used Logistic Regression in the SPSS (25.0) software to calculate the joint predictive value with the formula Logit(P) = − 12.62 − 1.466*Gal-9 + 1.249MELD, and plotted their joint ROC curve; with an AUC of 0.945, the joint curve significantly outperformed the individual curves (*p* < 0.05).

## Discussion

Acute decompensation, organ failure, and a high death rate are the hallmarks of acute deterioration of liver function in patients with chronic liver disease (ACLF). HBV-ACLF, caused by CHB and usually triggered by HBV activation, infection, or gastrointestinal bleeding, is the most prevalent type of ACLF in China. Therefore, identifying therapeutic targets for ACLF is crucial. It has been established that the galectin family member Gal-9 binds to Tim-3. Gal-9 binding to Tim-3 triggers immune inhibitory responses, such as cell apoptosis induction or exhaustion, regulating cytokine secretion^10^, and playing an important role in immune regulation. In theory, Gal-9 might be used as a therapeutic modulator to treat ACLF.

Recent research has demonstrated the immunomodulatory function of Gal-9 in various diseases. Gal-9 levels and their application in clinical practice as a biomarker may be crucial for tracking disease activity and encouraging individualized treatment choices^4^. As the ROC curve demonstrated that the predictive efficacy of Gal-9 was similar to that of the MELD score, Gal-9 may serve as a predictor of prognosis in patients with HBV-ACLF. There are few studies on Gal-9’s function in patients with liver failure, and little is known about its potential therapeutic benefits. The prognostic significance of Gal-9 in drug-induced liver injury-acute liver failure (DILI-ALF) was examined only by Rosen et al.^8^. According to their research, individuals with DILI-ALF who had high plasma levels of Gal-9 correlated positively with a risk of death, and those with Gal-9 levels over 389 pg/mL were more likely to develop systemic inflammatory response syndrome. In contrast, our research revealed a negative correlation between high Gal-9 levels and mortality risk in patients with HBV-ACLF, as well as a lower risk of death after 90 days in patients whose plasma Gal-9 levels were greater than 9.6 ng/mL. The discrepancy between our study results and those of Rosen et al. may be associated with differences in the etiology, type, or staging of liver failure. We examined the levels of Tim-3 and Gal-9 in the liver and peripheral blood tissues of 33 patients with HBV-ACLF and 33 with CHB We discovered that individuals with HBV-ACLF had greater plasma Gal-9 levels than patients with CHB. Furthermore, in contrast to the CHB group, the HBV-ACLF group exhibited higher expressions of the inflammatory cytokines linked to liver failure, such as IFN-γ and IL-6, with trends resembling those of Gal-9, indicating that Gal-9 may be a biological marker for HBV-ACLF.

To further understand the source of Gal-9 in patients with ACLF and evaluate the potential role of Gal-9 and its ligand Tim-3 in patients with HBV-ACLF, we used flow cytometry and found that Gal-9 and Tim-3 expressions were elevated in peripheral blood monocytes from CD8 + and CD4 + T cells of patients with HBV-ACLF. This suggests that Gal-9 may be involved in the immune response of patients with HBV-ACLF via T cells. Immunohistochemistry and double immunofluorescence staining showed that Gal-9 in patients with HBV-ACLF was localized in areas of active liver tissue regeneration and co-localized with CD68 + KCs in the liver tissue of patients with liver failure, indicating that KCs may primarily secrete Gal-9 in liver tissues. Research has indicated a relationship between elevated blood Gal-9 levels and liver fibrosis in patients with chronic liver disease, as seen by the association between these levels and the Fibrosis-4 and AST/PT ratio indices^11–13^. In this study, we created a correlation matrix heatmap using clinical characteristics associated with HBV-ACLF. We found that plasma Gal-9 levels had the strongest positive correlation with AST, with a correlation coefficient of 0.77, followed by a positive correlation coefficient of 0.66 with TBIL. The strongest negative correlation was with lactate, with a correlation coefficient of -0.94. AST and TBIL are crucial indicators of liver injury, and the positive relationship between Gal-9 and these indicators reflects the theoretical basis of Gal-9 as an ACLF biological marker. Lactate levels play a significant role in the prognosis of critically ill patients. Cardoso et al.^14^ found a significant correlation between baseline lactate levels and intensive care unit mortality, with an 8% increase in mortality for every 1-mmol/L increase in lactate levels. The APASL expert consensus on ACLF proposed that combining MELD and lactate to predict ACLF outcomes has higher specificity and sensitivity and better predictive value than using the MELD or CLIF-SOFA scores alone^9^, indicating a close relationship between lactate and disease prognosis. In our study, Gal-9 demonstrated a strong negative association with lactate levels, indirectly suggesting a potential association between variations in plasma Gal-9 concentrations and prognosis, consistent with the Kaplan–Meier survival curve results.

A meta-analysis^3^ focusing on the association between galactoglucan lectin levels and the risk of different liver diseases found that plasma Gal-9 levels were significantly higher in patients with hepatocellular carcinoma than in healthy controls; additionally, low Gal-9 expression predicted a worse prognosis in patients with hepatocellular carcinoma. This is similar to our findings in patients with ACLF. We found that plasma Gal-9 levels were significantly higher in the ACLF group compared to the CHB group, suggesting that the body secretes more Gal-9 in response to the severity of ACLF. The correlation between high plasma Gal-9 levels and a better prognosis in patients with ACLF also suggests that Gal-9 may have a “protective function”. However, the mechanism underlying the occurrence and development of Gal-9 in ACLF remains unclear.

In conclusion, this study identified a novel biomarker for HBV-ACLF, indicating that Gal-9 may aid in the clinical prognosis of HBV-ACLF. KCs may primarily secrete Gal-9 in liver tissues, and Gal-9 may play an immunomodulatory role in liver failure by engaging in immune regulation via CD8 + and CD4 + T cells. However, this study is limited by its small sample size and single-center design. Future studies will increase the sample size and focus on dynamic variations in Gal-9 levels in patients with ACLF to monitor disease progression and promote personalized treatment decisions.

## Electronic supplementary material

Below is the link to the electronic supplementary material.

Supplementary Material 1

## Data Availability

The data generated and/or analyzed during the current study are available with the corresponding authors upon reasonable request.
