# Supplementary material for: Galectin-9 as a new biomarker of acute-on-chronic liver failure
Source: Sci Rep. 2024 Sep 27;14:22303. doi: 10.1038/s41598-024-73397-6 (PMC11437140; doi:10.1038/s41598-024-73397-6)
Supplement: Supplementary file 1 — Supplementary Material 1 [file 41598_2024_73397_MOESM1_ESM.docx]

**Supplementary Information, Methods**

**Galectin-9 as a New Biomarker of Acute-on-Chronic Liver Failure**

*Scientific Reports*

**Jun Ling1, Shaoli You1, Weiwei Chen2, Xinxin Yang2, Yiwen Xv1, and Bing Zhu1***

1Hepatology Department, The Fifth Medical Center of Chinese PLA General Hospital, Beijing 10039, China

2Infectious Disease Department, The Fifth Medical Center of Chinese PLA General Hospital, Beijing 10039, China

***Corresponding author:**

The Fifth Medical Center of Chinese PLA General Hospital E-mail: zhubing302@163.com

**Peripheral blood mononuclear cell isolation**After centrifugation of 10 mL of whole blood at 2,000 rpm for 10 minutes, the plasma was aspirated and diluted by adding 6 mL of phosphate-buffered saline (PBS) solution and mixed well. Subsequently, two 15- mL centrifuge tubes of plasma were obtained, and 5 mL of Ficoll (P4350, Solarbio, Beijing, China) solution was added. The diluted blood was gently added to the upper layer of the Ficoll in the two centrifuge tubes and centrifuged at 2,500 rpm for 20 minutes. After centrifugation, the cell layer where the peripheral blood mononuclear cells were located turned white. The layer of cells was aspirated in another clean 15-mL centrifuge tube using a pipette, and 10–15 mL of PBS was added. We centrifuged the solution at 1,500 rpm for 10 minutes, removed the supernatant, and then added PBS and repeated the same procedure. After washing, we discarded the supernatant and froze the cells in 1 mL of serum-free cell freezing solution (HXJ6017, Huaxing Bio, Nei Mongol, China). The cryobox was placed in a refrigerator at -80°C overnight. On the next day, the cells were transferred to liquid nitrogen for long-term storage.

**Immunohistochemistry (IHC) staining**

Paraffin-embedded wax blocks of the liver tissue from patients with acute-on-chronic liver failure (ACLF) and chronic hepatitis B were cut into 4-μm thick blocks and placed on slides. Then, the sections were stained with IHC to assess galectin-9 (Gal-9) expression. Slides were dewaxed in xylene and rehydrated through a graded ethanol series (100 %, 95 %, 90 %, 80 %, and 70 %); next, antigen repair was performed in

sodium citrate buffer (0.1 mol/L of citric acid and 0.1 mol/L of sodium citrate; pH 6.0) at high temperature and pressure for 90 seconds. An appropriate amount of endogenous peroxidase blocker was added, the sample was incubated for 10 minutes at room temperature, and then it was rinsed thrice in PBS buffer for 3 minutes. Gal-9 antibody (1:400; ab69630, Abcam, Cambridge, UK) was added and incubated for 60 minutes at 37 °C; the slides were rinsed thrice in PBS buffer for 3 minutes. Subsequently, 100 μL of reaction enhancement solution was added dropwise and incubated at 37 °C for 20 minutes; the slides were rinsed thrice in PBS buffer for 3 minutes. Furthermore, 100 μL of Enhanced Enzyme Labeled Goat Anti-Rabbit Immunoglobulin (Ig)-G Polymer was added dropwise and incubated at 37 °C for 20 minutes, and the slides were rinsed thrice in PBS buffer for 3 minutes (endogenous peroxidase blocker, Reaction Enhancement Solution, and Enhanced Enzyme Labeled Goat Anti-Rabbit IgG Polymer; Rabbit 2-Step Assay Kit PV-9001; ZSGB-BIO, Beijing, China). Diaminobenzidine (PV-9001; ZSGB- BIO) was applied for color development, incubated for 5–8 minutes at room temperature, and then, the slides were rinsed in tap water to stop further color development. The slides were restained with hematoxylin for 5 minutes, rinsed in running water, dehydrated in a gradient of ethanol (70 %, 80 %, 90 %, 95 %, and 100 %), treated with xylene, and sealed. Slides were digitally scanned using a fluorescent multicolor scanner set (Leica, Inc., Teaneck, NJ) at ×20.

**Immunofluorescent staining**

Paraffin-embedded ACLF tissue sections were prepared using the above procedure. The sections were deparaffinised, rehydrated, microwave repaired for antigen and closed using the Multiplex Fluorescence Immunohistochemistry Kit-Four Color TSA-RM-275 (10001100020, PANOVUE, China). Then, the slides were incubated with the anti-Gal-9 antibodie in a humidified room at 4 ℃ overnight. The slides were washed three times with PBS and then incubated with HRP-labelled goat anti-rabbit Ig-G secondary antibody (10001100020, PANOVUE, China) for 1 hr at 4 ℃ under light protection, followed by fluorescent staining to amplify the signals and then microwave repair to reduce the noise, and then washed to end the single staining. Additional CD68 staining was continued from closure by incubating CD68 primary and secondary antibodies, again using fluorescent staining to amplify the signal, followed by microwave repair to reduce the noise, and finally nuclear staining and sealing with an antifluorescent sealer (ZU9557; ZSGB-BIO), and then observing the samples and imaging them under a fluorescence multicolour scanner (Aperio Versa 8; Leica, Inc.). imaging. Antibodies used for immunofluorescence staining were anti-Gal-9 (1:400; ab69630; Abcam) and anti-CD68 antibody (1:800, ab283654; Abcam).
